# Peer‐assisted learning in scenario‐based simulation

**DOI:** 10.1111/medu.13563

**Published:** 2018-03-24

**Authors:** Leo Nunnink, Andrea Thompson

## What problems were addressed?

Although scenario‐based simulation is an established learning method, some potential benefits remain unexplored. These include its provision of unique opportunities for students to acquire the perspective of the patient and those of interprofessional colleagues. Simulation may also facilitate a detailed understanding of pathophysiology through the writing of scenarios. We sought to extend the potential of simulation by asking students to write, deliver and debrief simulation scenarios.

## What was tried?

During an established 2‐day, scenario‐based programme using a manikin patient (Laerdal SimMan), final‐year medical students were asked to write, deliver and debrief a simulation scenario while working in groups.

Students were encouraged to base their scenario on any clinical case they had encountered. They were given a standard template with headings for learning objectives, patient details, required learner actions and simulated patient responses to those actions.

Students had 1 month to prepare a patient history, script for the patient, hospital medical record, and relevant blood test and radiology results. Preparation required thorough research of the pathophysiology and clinical evolution of the chosen condition, as well as expected responses to pharmacological interventions.

Scenarios underwent expert review at an early stage of development and again immediately prior to delivery. Scenarios were delivered by students (‘instructor’ peers), with one student ‘acting’ as the voice of the patient (audible from the manikin's mouth) and others acting as nurses. Another group of students functioned as scenario participants (‘learner’ peers), assessing and treating the simulated patient. Each scenario was followed by an educational debriefing, facilitated by the ‘instructors’. A total of 79 students participated. Groups took turns in ‘instructing’ and ‘learning’, without prior knowledge of the other groups’ scenarios.

## What lessons were learned?

The delivery of peer‐written and peer‐run simulation scenarios was feasible, although it represented a demanding exercise for the simulation faculty staff. Unanticipated actions on the part of both the student participants in the scenario and the student instructors delivering the scenario required flexibility and spontaneity.

Expert review of scenarios prior to delivery was a key factor in successful delivery as students tended to write scenarios that expected an unrealistically high level of performance from their peers.

Students were able to perform high‐standard educational debriefing. Preparing for technical debriefing motivated students to research best‐practice management guidelines for their chosen condition. With minimal teaching students were able to provide their peers with feedback on non‐technical behaviours (teamwork and communication) based solely on exposure to previous instructor‐led debriefings.

All simulation scenarios are evaluated for quality assurance. For the peer‐led simulation exercises, students were asked whether the components of developing, delivering and debriefing the scenario improved their knowledge; students agreed in 94%, 91% and 96% of responses, respectively. Students (*n* = 79) gave the exercise an overall mean rating of 4.6 on a scale of 1–5 (1 = unsatisfactory, 5 = excellent).

Qualitative feedback from students suggested potential benefits not foreseen by the instructors, which are now the subject of an ongoing research project. These included better understanding of the patient's perspective acquired by acting as the voice of the ‘patient’ and improved interprofessional understanding achieved through acting as a nurse.

